# Handwritten mathematical symbols dataset

**DOI:** 10.1016/j.dib.2016.02.060

**Published:** 2016-03-02

**Authors:** Yassine Chajri, Belaid Bouikhalene

**Affiliations:** Laboratory of Information Processing and Decision Support, USMS, Beni Mellal, Morocco

**Keywords:** Image processing, Handwritten mathematical symbols, Documents, Recognition

## Abstract

Due to the technological advances in recent years, paper scientific documents are used less and less. Thus, the trend in the scientific community to use digital documents has increased considerably. Among these documents, there are scientific documents and more specifically mathematics documents.

In this context, we present our own dataset of handwritten mathematical symbols composed of 10,379 images.

This dataset gathers Arabic characters, Latin characters, Arabic numerals, Latin numerals, arithmetic operators, set-symbols, comparison symbols, delimiters, etc.

**Specifications Table**

**Table:** 

Subject area	*Computer science*
More specific subject area	*Image processing, handwritten mathematical symbols, documents recognition*
Type of data	*Image*
How data was acquired	*Handwritten, Scanner, Marker*
Data format	*Jpeg image*
Experimental factors	*We asked 97 students of our university to write a list of mathematical symbols, we used an HP G3110 to scan data and we used a marker in symbols writing*
Experimental features	*10,379 Images with a size of 80×60 pixels*
Data source location	*Beni Mellal, Morocco*
Data accessibility	*Within this article*

**Value of the data**

• Given the importance of mathematics in all branches of science (physics, engineering, medicine, economics, etc.), the recognition of handwritten mathematical expressions has become a very important area of scientific research.
• We prepared a dataset which contains 10,379 symbols written in marker and which represents the most frequently used symbols.
• This dataset gathers Arabic and Latin symbols which make it a very important dataset compared to the others presented in the literature.
• It contains a large number of mathematical symbols and is characterized by several styles of writing.
• This dataset is very useful to implement a recognition system for handwritten mathematical documents and it will help facilitate the research in this important area.

## 1 Data, experimental design, materials and methods

### 1.1 Data preparation

**Fig. 1:**
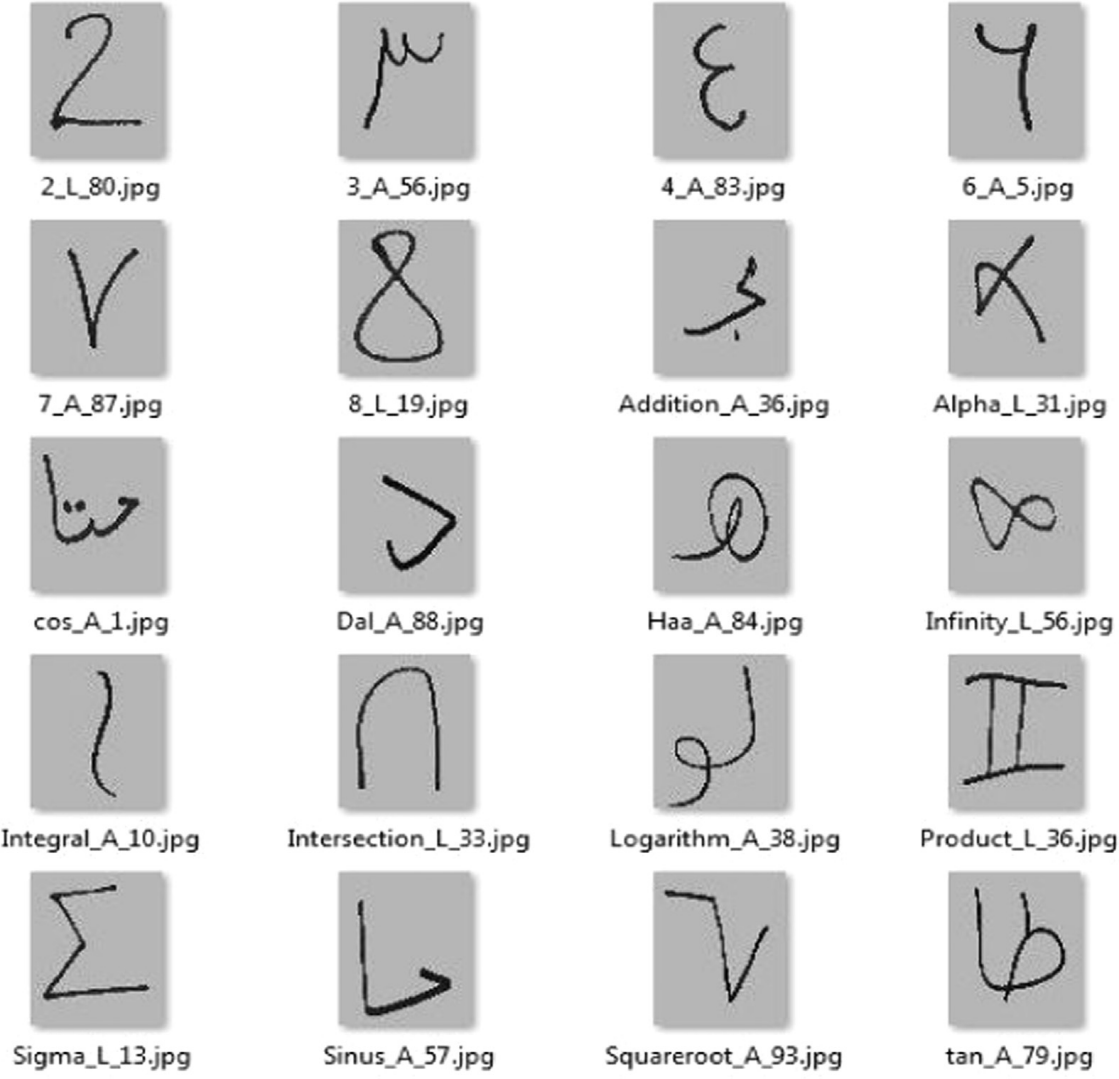
Examples of handwritten mathematical symbols in our dataset.

For the preparation of our dataset we;

• Targeted 97 students (47 male and 50 female) of our university (Bachelor, Master and Doctorate).
• Asked them to write a list of mathematical symbols in order to have a diversity of writing styles.
• Used an HP G3110 to scan pages.
• Used Radon transform [1], [2], [3] for skew detection and correction.
• Used histogram equalization [4] for images normalization.
• Median filtering [5], [6] for image noise reduction.
• Used connected components algorithm for symbols detection [7].
• Extracted 10,379 sub-images with a size of 80*60 which contain the symbols (Fig.1).

The images are named in three parts:

• The first is the symbol name.
• The second part makes the difference between Arabic and Latin symbols (A or L).
• The last part is represented by numbers from 1 to 97 (Table 1, Table 2, Table 3, Table 4).

## Figures and Tables

**Table 1:** Mathematical symbols dataset.

**Symbols**	**Description**
**A, B, C, D, E, F, G, H, I,…………….,U, V, W, X, Y, Z**	**Latin characters**
**م،ن،ه،و،ي،ء،……………………..ا،ب،ت،ث،ج**	**Arabic characters**
**1,2,3,4,5,6,7,8,9**	**Latin numerals**
**٠,١,٢,٣,_,_,_,٧,٨,٩**	**Arabic numerals**
**∑**,**∏**,	**summation or product symbols**
**∫**	**Integral symbol**
**√**	**Square root**
**|, (,), {, }, [, ]**	**Delimiters symbols**
=, ≠, <, >,,+, *, ×, /,,←,⋂, ⋃, ⊃, ⊄, ⊂, ∈, ∉	**Arithmetic operators, comparison operators, set symbols**

**Table 2:** Comparison between the Arabic and Latin characters.

**Latin characters**	**Arabic characters**
A	ا
B	ب
C	ت
D	ث
E	ج
F	ح
G	خ
H	د
I	ذ
J	ر
K	ز
L	س
M	ش
N	ص
O	ض
P	ط
Q	ظ
R	ع
S	غ
T	ف
U	ق
V	ك
W	ل
X	م
Y	ن
Z	ه
_	و
_	ي
	ء

**Table 3:** Comparison between the Arabic and Latin numerals.

**Latin numerals**	**Arabic numerals**
0	٠
1	١
2	٢
3	٣
4	٤
5	٥
6	٦
7	٧
8	٨
9	٩

**Table 4:** Some of the composed symbols.

**Composed Latin symbols**	**Composed Arabic symbols**
**Cos**	**حتا**
**Sin**	**حا**
**Tan**	**طا**
**Log**	**لو**
**Lim**	**نها**
**….**	**….**
